# Atomevo: a web server combining protein modelling, docking, molecular dynamic simulation and MMPBSA analysis of *Candida antarctica* lipase B (CalB) fusion protein

**DOI:** 10.1186/s40643-022-00546-y

**Published:** 2022-05-13

**Authors:** Jin-Heng Hao, Dun-Jin Zheng, Yu-Hao Ye, Jie-Ting Yu, Xin-Yao Li, Mei-Jie Xiong, Wen-Hao Jiang, Kang-Ping He, Pei-Yu Li, Yong-Si Lv, Wei-Ming Gu, Lin-Hao Lai, Yi-Da Wu, Shi-Lin Cao

**Affiliations:** 1 Guangdong Key Laboratory of Food Intelligent Manufacturing, School of Food Science and Technology, Foshan University, Foshan, 528000 China; 2 School of Food Science and Technology, Foshan University, Foshan, 528225 Guangdong China; 3 School of Food Science, South China Agricultural University, Guangzhou, 510642 China

**Keywords:** Biofuel, Molecular dynamics simulation, Docking, MMPBSA, Web Server

## Abstract

Although current computational biology software is available and has prompted the development of enzyme–substrates simulation, they are difficult to install and inconvenient to use. This makes the time-consuming and error-prone process. By far there is still a lack of a complete tool which can provide a one-stop service for the enzyme–substrates simulation process. Hence, in this study, several computational biology software was extended development and integrated as a website toolbox named Atomevo. The Atomevo is a free web server providing a user-friendly interface for enzyme–substrates simulation: (1) protein homologous modeling; (2) parallel docking module of Autodock Vina 1.2; (3) automatic modeling builder for Gromacs molecular dynamics simulation package; and (4) Molecular Mechanics/Poisson–Boltzmann Surface Area (MMPBSA) analysis module for receptor–ligand binding affinity analysis. We officially launched the web server and provided instructions through a case for the design and simulation of *Candida antarctica* lipase B (CalB) fusion protein called Maltose Binding Protein—Thioredoxin A—*Candida antarctica* lipase B (MBP-TrxA-CalB).

**Graphical Abstract:**

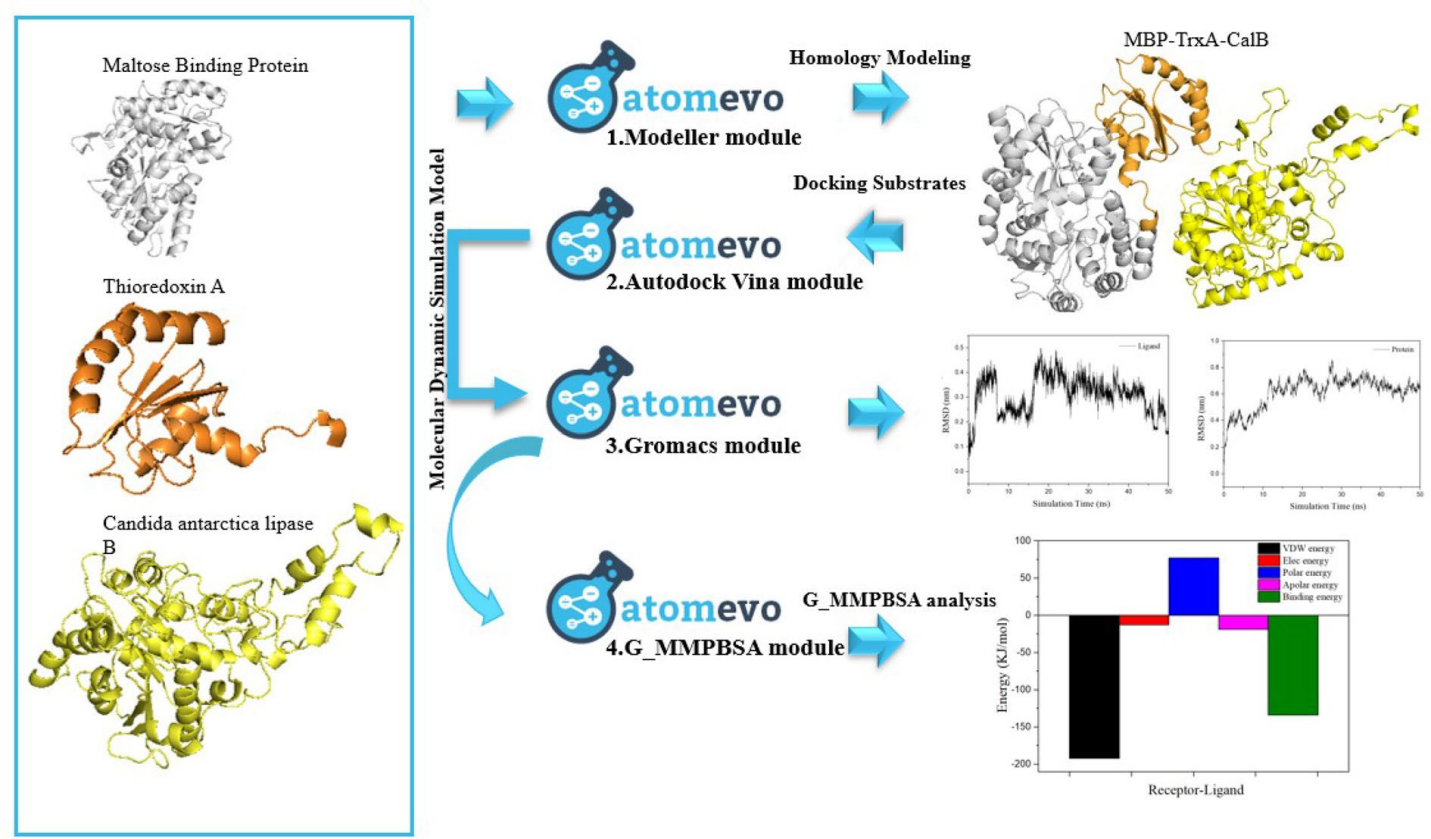

**Supplementary Information:**

The online version contains supplementary material available at 10.1186/s40643-022-00546-y.

## Introduction

Enzymes, such as lipase, exhibit great potential in bioenergy and biomedicine are widely used for biofuel production (Fatma et al. 2021). However, there are increasing demand of efficient enzymes for practical application. During the past decades, an increasing number of computational methods (Zhang et al. 2020; Xu et al. 2021) and software have been developed and exhibit great potential in the investigation of the structure and function relationship of enzymes (Rester 2008; Baek et al. 2021; Jumper et al. 2021) and rational and semirational design of enzyme catalysts (Liu et al. 2021). Homology modelling, molecular docking (Eberhardt et al. 2021) and molecular dynamics simulation (Phillips et al. 2020) are three important computational biology methods (McGregor et al. 2007).

For example, a typical investigation towards enzyme–substrates interaction includes the following processes: (1) building the protein structure; (2) docking of candidate substrate molecules into the enzyme potential catalytic site and estimating rank the binding affinity between the substrate and enzyme to obtain the potential drug candidates; (3) performing the molecular dynamics (MD) simulation between the substrate and enzyme, according to the docking results; and (4) performing the Molecular Mechanics/Poisson–Boltzmann Surface Area (MMPBSA) analysis to further confirm the enzyme/substrate interaction. Up to now, several molecular docking AutoDock-Vina (Eberhardt et al. 2021), Ledock (Wang et al. 2016), SwissDock (Grosdidier et al. 2011), Schrödinger (Bhachoo and Beuming 2017), etc.) and molecular dynamic simulation (gromacs (Berendsen et al. 1995), NAMD (Nelson et al. 1996; Phillips et al. 2020), lammps (Thompson et al. 2022)) software have been developed. However, most software need to be run on the Linux command-line system, which raises the bar of the users’ software debugging and installation capabilities. Although some software such as SwissDock (Grosdidier et al. 2011) can perform online docking tasks through web servers on the window, it can only provide a single molecular docking service and cannot complete a whole set of virtual screening. DS (Valentine et al. 2021) and Schrödinger (Bhachoo and Beuming 2017) can be used under windows operation system. They are convenient and simple in interface, but they are costly and thus not accessible to students or laboratories with insufficient funds. Thus, a free web server that providing molecular docking, molecular dynamic simulation, and relative data analysis is a great tool making enzyme rational and semirational design more accessible to the scientific community.

Herein, several academic free software for predicting the protein structure, enzyme–substrate molecular docking, and molecular dynamic simulation were extended development. The Atomevo web server is a free web server providing a user-friendly interface for (1) protein homologous (Modelling Module); (2) parallel docking module of Autodock Vina (Vina Module); (3) automatic modeling builder for Gromacs molecular dynamics simulation package (GMX Module); and (4) Molecular Mechanics Poisson–Boltzmann Surface Area (MMPBSA) analysis module for receptor–ligand binding affinity analysis (G_MMPBSA Module).

In this study, we officially launched the web server and provided instructions through a case description for (1) structure modelling of the *Candida antarctica* lipase B (CalB) fusion protein (MBP-TrxA-CalB), which fused with maltose binding protein and thioredoxin A (TrxA) to improve the CalB expression level in *Escherichia coli* (*E. coli*) by Atomevo-Modelling Module; (2) molecular docking between MBP-TrxA-CalB (MT-CalB) and fatty acid small molecules by Atomevo-Vina module; (3) molecular dynamic simulation of MT-CalB/fatty acid complex via Atomevo-GMX module; and (4) MMPBSA analysis between MT-CalB and fatty acid via Atomevo-G_MMPBSA module. Finally, the MT-CalB was expressed in *E. coli* and the enzyme properties were characterized. Generally speaking, this platform provides a user-friendly, comprehensive, and flexible tool for protein modelling, docking, molecular dynamic simulation to facilitate in the exploration of enzyme (such as lipase) structure and function relationships, as well as the rational and semi-rational design of enzyme catalysts.

## Materials and methods

### The program and modules used in Atomevo platform

The modules and software used in this platform are all open source projects or academic free software, and the modules are secondary-developed. The modules used mainly include: (1) molecular docking software: AutoDock Vina (Linux 1.2 version, open source project) (Eberhardt et al. 2021); molecular Docking scoring software XScore (academic free, non-open source project) (Wang et al. 2002) protein–ligand interaction analysis tool Protein–Ligand Interaction Profiler (academic free, open source project, https://github.com/ssalentin/plip) (Salentin et al. 2015); chemical toolbox openbabel (open source project, https://github.com/openbabel/openbabel) (O'Boyle et al. 2011); GROMACS2019.1. (open source project) (Berendsen et al. 1995).

### Implementation of Atomevo platform

The Atomevo web server consists of the user interface, the local server and the job backend. The user interface is implemented in https://atomevo.com. This interface allows user to upload the input files and the configure parameters (or files), as well as download the output files. The normal server is deployed in Ali Cloud Server and the advance server is deployed in our local server with Intel(R) Xeon(R) CPU E5-2690 v3, MSI RTX 2080 with 8 GB of RAM, MD1200 storage devices 72 TB and 64 GB RAM.

The job backend is developed using PHP technology. Thinkphp framework is used as a backend development framework and Medoo as a database framework. Axios is used to send the request to the background and Workerman is used to build the instant messaging framework.Vue.js is used to build progressive framework and Wepack module loader is used to convert the dependent module to static files which represents these packages. PhpSpreadsheet, a plug-in, is used to score statistics, and Handsontable, a tabular interactive plug-in in the footer, is used to organize, count, calculate and summarize the tables.

### Implementation of protein homologous modeller module

We have created the Modeller module on the Atomevo platform to perform Homology Modeling online (https://atomevo.com/modeller).

**Fig. 1:**
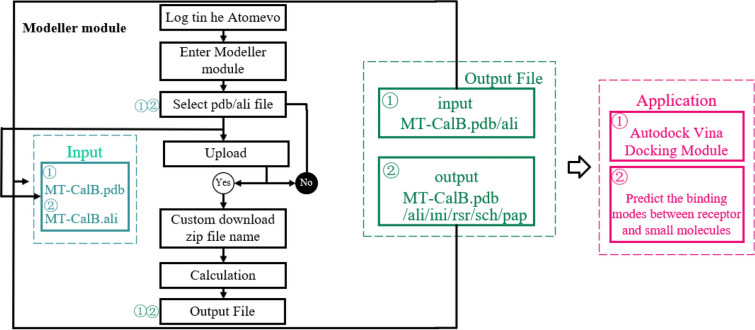
Flow diagram of modeller module

The algorithm of the Modeller module includes several steps (Fig. 1): (1) input files (*.pdb, *.ali) for Modeller single-template modeling module calculation; (2) performing the Modeller calculation and obtaining the calculation results file (*.pdb, *.ali, *.ini, *.rsr, *.sch and *.pap files); and (3) output files and provide them to users for download.

The Modeller module is divided into three sub-modules: single-template modelling module, multi-template modelling module, and sequence alignment module. Its workflow includes the following steps (take a single-template modelling module as an example): (1) the user uploads the hydrogenated pdb file and the template ali file and submits a calculation task to the server; (2) after receiving the calculation request submitted by the user, the server creates a new calculation process and calls the calculation script of the Modeller single-template modelling module to calculation for protein homologous; and (3) when the calculation is completed, the server terminates the calculation process and moves the output file to a folder named after the task number, packs the folder, and sends the calculation file and task completion reminder mail to the user through the phpmailer module.

### Implementation of the molecular docking module

To perform virtual screening of lipase by parallel module of Autodock Vina online, we have established the Autodock Vina module on the platform (https://atomevo.com/autodock-vina).

The Autodock Vina module includes two parts: Vina docking module and result analysis module.

**Fig. 2:**
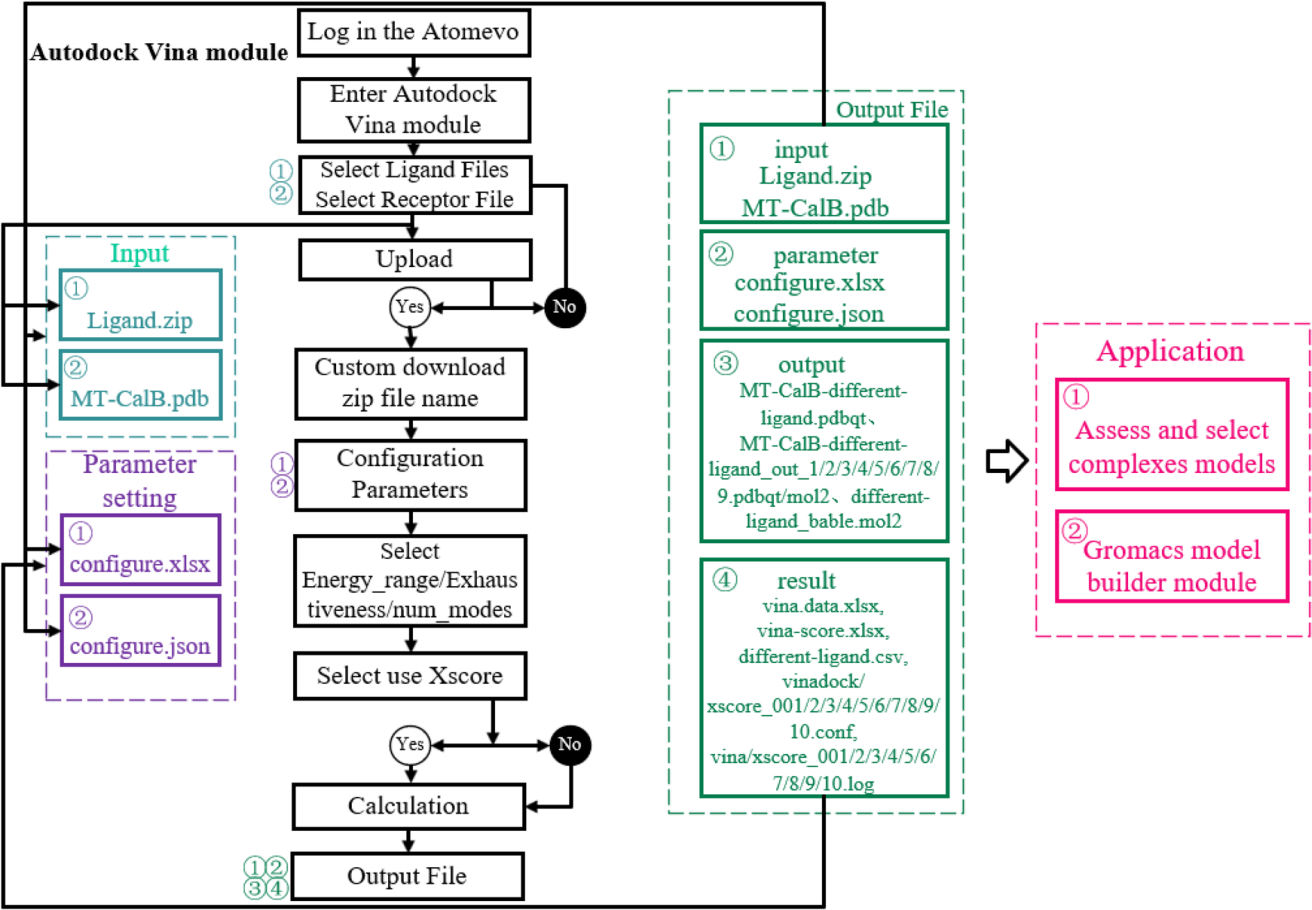
Flow diagram of autodock vina module

The algorithm of the Autodock Vina module includes several steps (Fig. 2): (1) creating the vina configure file (conf.txt) by reading the parameters in the configure file (*.xlsx); (2) converting input file (*.pdb) to *.pdbqt; (3) making separate directories for AutoDock vina calculation; (4) running docking (vina –configure vinadock.conf); and (5) analyzing the result.

Results analysis module is showed as follows:

(1) Vina result analysis: the multimodel pdbqt files (*_out.pdbqt) are split into individual models files (*_out*.pdbqt) using the vina_split command. The docking result of each individual model (including affinity (kcal/mol), dist from rmsd rmsd l.b., best mode rmsd u.b.) is exported through phpexcel plug-in into the vina-score file (vina-score.xlsx).

(2) X-Score analysis: first, the individual models files (*_out*.pdbqt) are converted to *_out*.mol2 by openbabel software; second, the ligand files (*_out*.mol2) and the receptor files (*.pdb) are submitted to the X-Score software (Wang et al. 2002). X-Score is a “Scoring function”, which has its major applications to structure-based drug design studies. It computes a binding score for a given protein–ligand complex structure, and this binding score correlates to experimental binding constants well. Three individual empirical scoring results, including HPScore, HMScore, and HSScore are exported through phpexcel plug-in into the ledock-score file (ledock-score.xlsx).

The computing platform is divided into Molecular preprocessing module, docking computing module, Grading module, and protein–ligand interaction analysis module.

The molecular preprocessing module mainly consists of the Openbabel module. Its workflow includes the following steps: (1) the user sends a compute request to the background through Axios from the client. Then the structure file needed to be converted should be uploaded to the server. And then, the user can select the output file type and output settings. (2) The Thinkphp framework is called the Openbabel software for calculation when the server receives the request. And finally, the server tidies up the compressed output file through Linux's compressed packaging commands.

The Docking computing module's workflow includes several steps: (1) the client is used to submit computational requests through the network interface and upload the relevant ligands, receptor structures, and docking condition profiles; (2) when the server receives the request, the server runs the modules of AutoDock Vina for docking calculation; and (3) the Linux operating system's file consolidation functions are used to settle the input file, output parameter file and pigeonhole to Input file, Output file, Parameter and Result separately.

The implementation method of molecular docking parallel computing: split the execution task of autodock_vina.php and create the Child process file named autodock_vina_chind.php. The Child process is in charge of the calculating and scoring operations between a single ligand and receptor. The result will be stored in the Redis. The calculation between a ligand and a receptor is performed in this file. When a new task is received, autodock_vina.php will use Popen (a php function) to perform autodock_vinachind.php subprocess file and return a file pointer to Parent process autodock_vina.php. The while loop condition formulation is used to wait for the end of Child process, and Fget (a php function) is used to determine if it is false and Pclose (a php function) is used to close all the completed subprocess files. Then the Child process continues to open until all the tasks in the configure.xlsx are completed. When all the Child processes are completed, the Parent process reads the data in Redis module to perform a series of operations, such as summarizing the data.

The Grading module's workflow includes several steps: (1) the Grading module invokes the calculated results of the docking ligands (which requires mol2 format, otherwise use openbabel for conversion) and docking receptor file (which need pdb format, otherwise use openbabel for conversion) in the computing platform automatically; (2) the XScore is invoked to calculate through the Thinkphp framework and grade the results; and (3) the PhpSpreadsheet and Handsontable plug-in are invoked to summarize, sort out and output the docking scoring results of each group of docking calculations which is from XScore score result and the corresponding calculation module.

### Molecular dynamic simulation detail for MT-CalB using Gromacs module

To exam the conformational stability of the receptor–ligand complex, the Gromacs model builder module is deployed on the Atomevo platform (https://atomevo.com/gromacs).

**Fig. 3:**
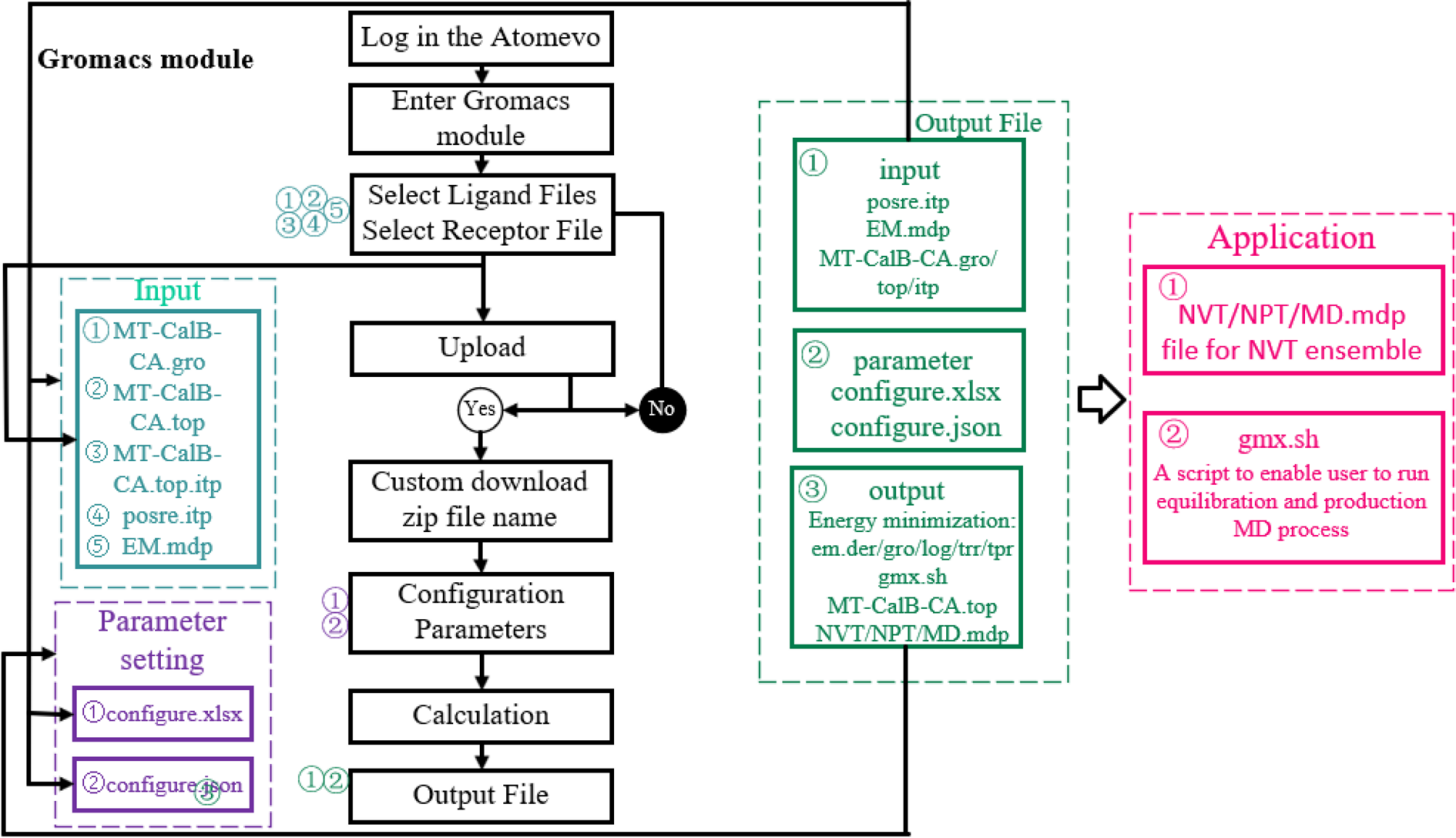
Flow diagram of Gromacs model builder module

The algorithm detail of the Gromacs_model_builder module includes several steps (Fig. 3): (1) creating the Gromacs_model_builder configure file by reading the parameters in the configure.file (*.xlsx); (2) inputing files (*.gro, *.top, *.itp and *.mdp) for Gromacs_model_builder calculation; (3) performing the define box and dolvate, add ions and energy minimization calculation; (4) generating NVT.mdp, NPT.mdp and MD.mpt file for NVT ensemble, NPT ensemble and MD simulation; and (5) generating a shell script to enable user to run equilibration and production MD process in their own computer.

In this research, the MT-CalB with Chaulmoogric Acid (CA) ligands was placed into the center of a cubic, in which the MT-CalB–ligand complex was 1.5 nm apart from the box margin (with the size of 15.302 11.055 8.518 nm). Then, 15 Na + were added to the box to balance the electrical neutrality of the simulation system.

First, the simulation box was submitted to 50,000 steps of energy minimization (EM) at 303.15 K using Particle-Mesh Ewald method. Then, position-restrained MD simulation was performed to equilibrate the solvent and ions around the ligand–receptor molecule via NVT ensemble (constant Number of particles, Volume, and Temperature), following by NPT ensemble (constant Number of particles, Pressure, and Temperature) and 50 ns production MD.

### Implementation of the G_MMPBSA module

To execute g_mmpbsa calculations on the assembly model of receptor and ligands online, we have established the g_mmpbsa module on the Atomevo (https://atomevo.com/g-mmpbsa).

**Fig. 4:**
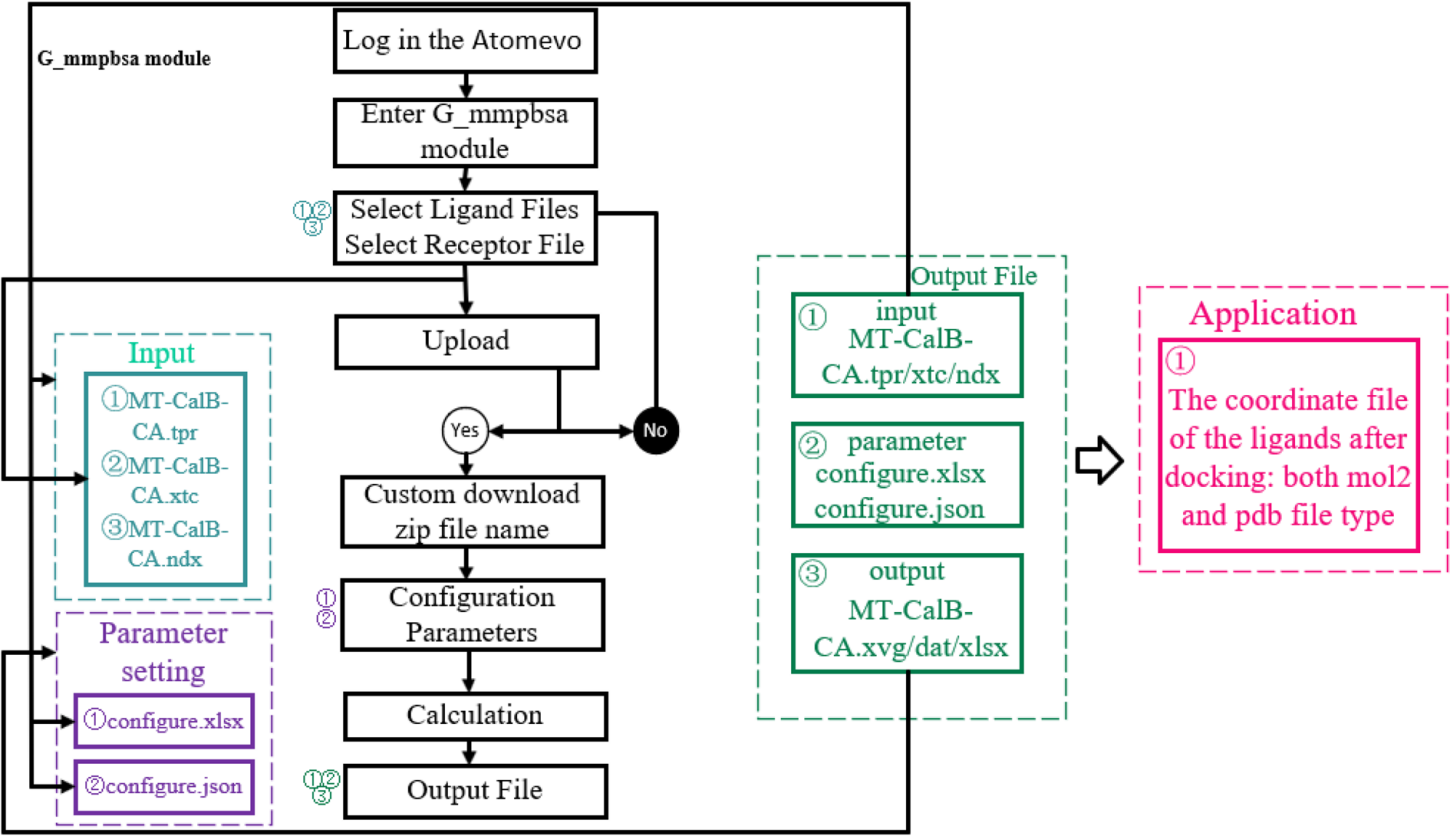
Flow diagram of g_mmpbsa module

Algorithm details: g_mmpbsa module includes several steps (Fig. 4): (1) creating the g_mmpbsa configure.file by reading the parameters in the configure.file (*.xlsx); (2) inputing files (*.tpr, *.xtc, and *.ndx) for g_mmpbsa calculation; (3) performing the g_mmpbsa calculation and obtaining the calculation results file (*.xvg and *.dat files); and (4) analysing the result *.xvg and *.dat files by performing with two python scripts (MmPbSaStat.py and MmPbSaDecomp.py) and the results are exported through phpexcel plug-in into the ledock-score file (g_mmpbsa_analysis.xlsx).

The Molecular mechanics Poisson–Boltzmann surface area (MMPBSA) analysis was performed by g_mmpbsa tool (Kumari et al. 2014). This paper analyzed the last 10 ns of the MT-CalB models that has been balanced.

### Experimental verification of design results for MT-CalB

The DNA sequence which encoding the Mycobacterium smegmatis porin A (mSpA) signal peptide was fused with both Maltose Binding Protein (MBP) and TrxA domains at the N-terminal of CalB domain, and cloned into the expression vector pETDuet-1 simultaneously to form the recombinant plasmid mSpA-MT-CalB-pETDuet-1. The *E. coli* strain containing mSpA-MT-CalB-pETDuet-1 was inoculated in the Luria–Bertani (LB) fermentation medium and incubated at 37 °C with shaking at 180 rpm, then 0.3 mM of Isopropyl β-d-1-thiogalactopyranoside (IPTG) inducer was added, while OD reached 0.6. Then the fermentation was performed at 15 °C for 30 h. The MT-CalB protein was extracted from the bacterial periplasmic space. The lipase activity could be detected as follows: a given amount of MT-CalB protein was first mixed with 2 mL phosphate buffered saline solution (50 mM, pH 7.0) at 30 °C, and then 1 mL *N*-α-benzoyl d, l-arginine-pyritroaniline solution (3.679 mM) was added to start the reaction. Finally, 5.3 mL anhydrous ethanol was added to terminate the reaction. The release amount of *p*-nitroaniline (pNA) can be determined by 405 nm spectrophotometry.

## Results

### Design of MT-CalB sequence

We fused the solution-promoting label MBP at the N-terminal for increasing the soluble expression of the lipase. In addition, the TrxA label, which greatly improved proper disulfide bond formation (Manta et al. 2019), was followed by located between the C-terminal of MBP label and N-terminal of the CalB domain.

### Homology modeling of MT-CalB structure using Atomevo modeller module

**Fig. 5:**
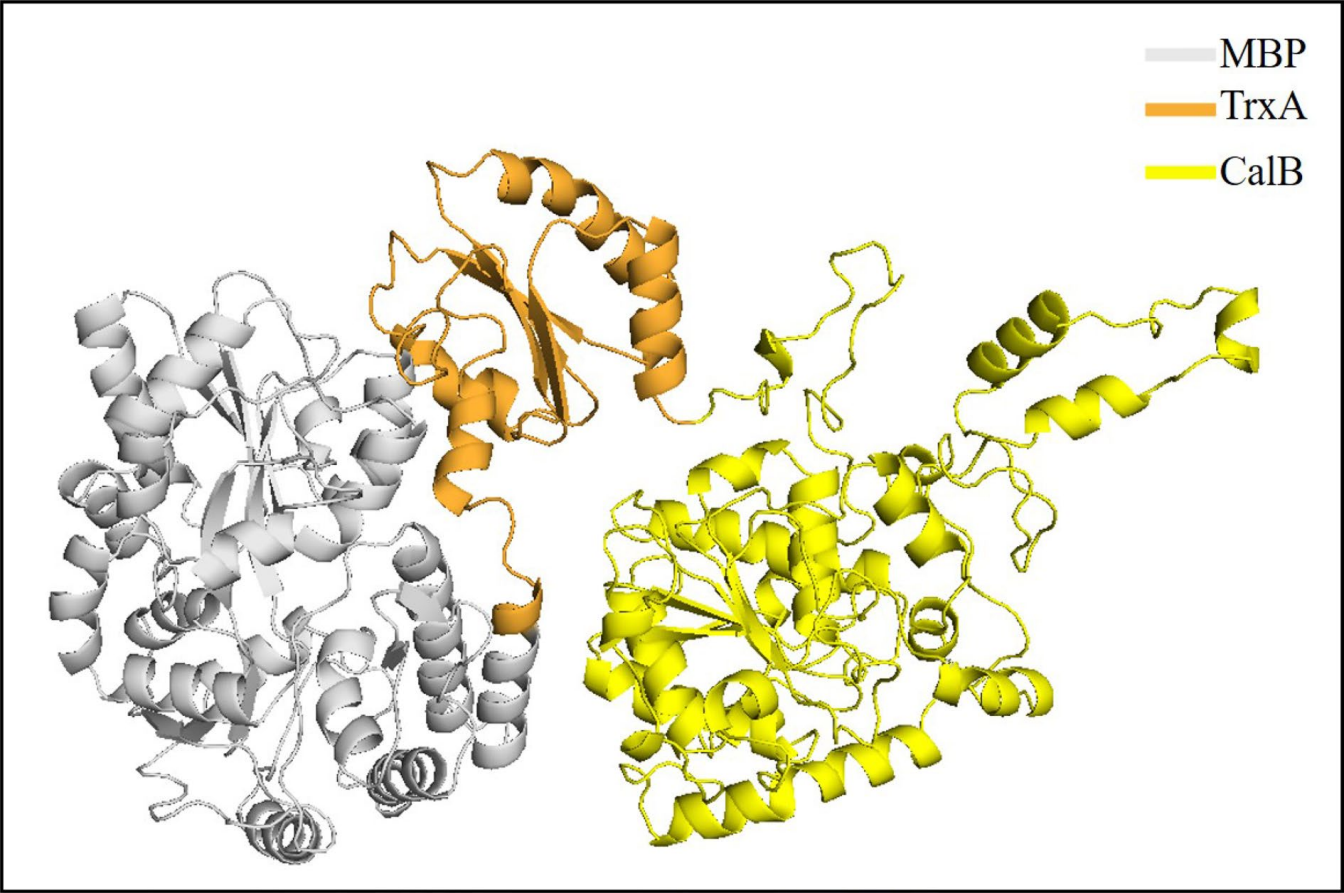
3D structure of MT-CalB lipase

**Fig. 6:**
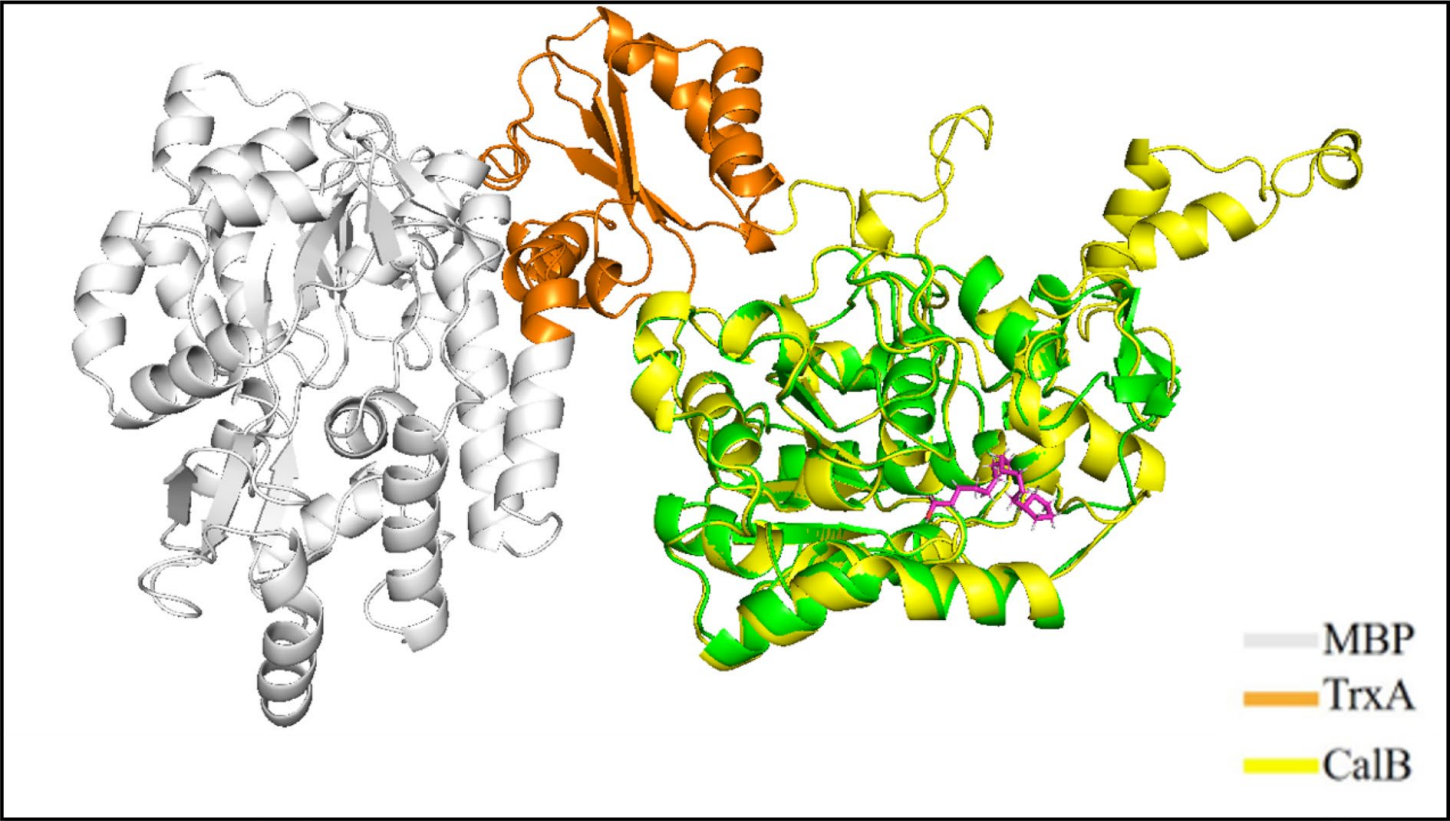
Comparation of MT-CalB and CalB

In this example, The MT-CalB structure was designed to increase the CalB expression level in *E. coli* using the Modeller module. The protein sequence of the whole lipase of MT-CalB is represented in Table S1. In addition, the 3D structure of MT-CalB is displayed in Fig. 5, and the MBP, TrxA, and CalB domain are colored in gray, orange, and yellow, respectively. By comparing with homologous modeling CalB (Fig. 6), it was found that MT-CalB and CalB basically overlapped, the active pocket of MT-CalB was maintained, MBP and TrxA were far away from the active center of CalB, and the overall structure of CalB was not affected.

### Docking of MT-CalB and substrates using Atomevo Autodock Vina module

In this example, the MT-CalB was docking with ten fatty acid ligands. Ligands database preparation for docking lipase (MT-CalB) was obtained from PubChem (Additional file 1: Table S2).

**Fig. 7:**
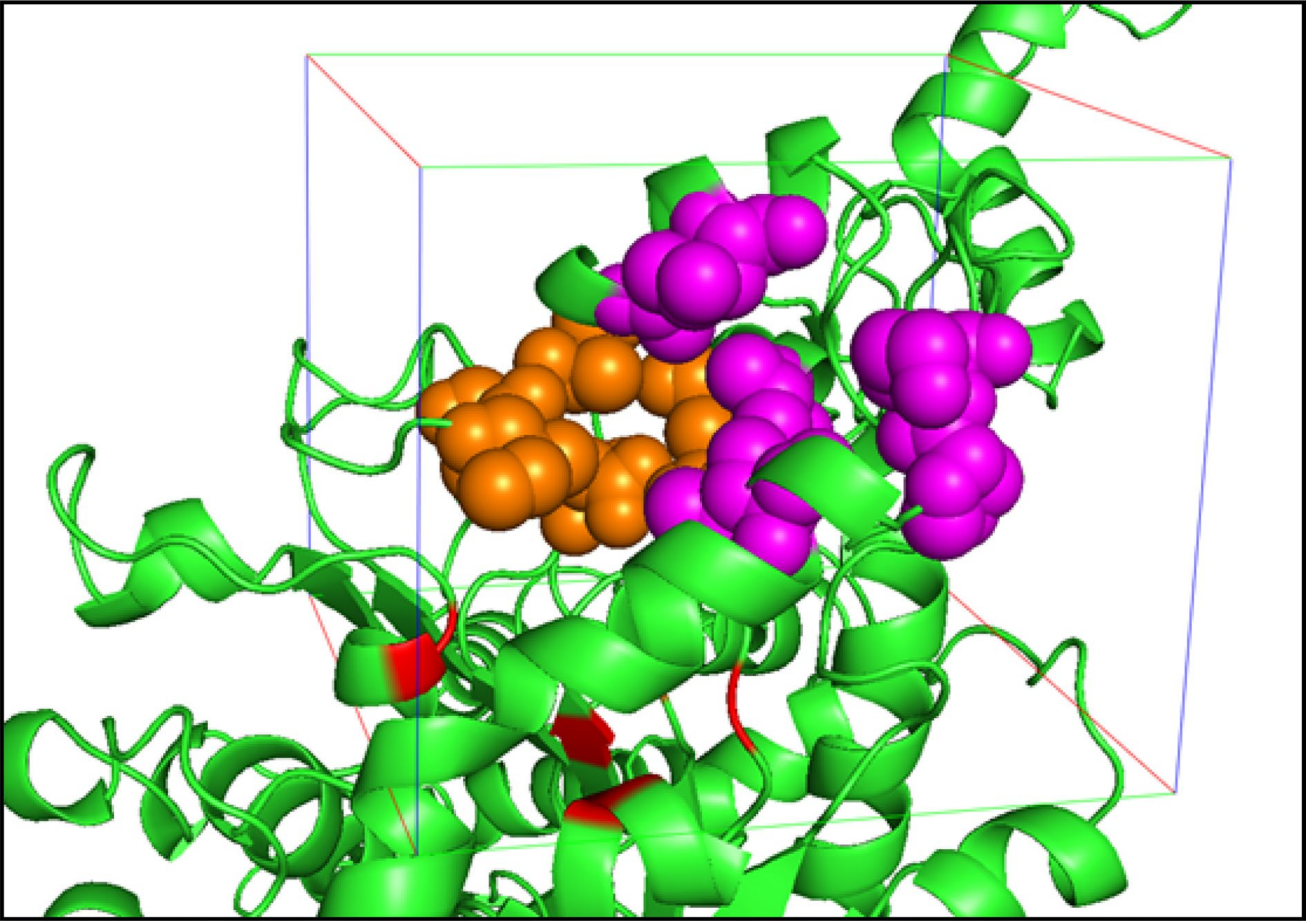
Molecular docking pocket of MT-CalB

The input files pdb-ligands.zip (including *.pdb files for both receptor and ligands can be submitted by uploading relative files to the system (Additional file 1: Fig. S1A), and the configure parameters can be submitted by uploading configure file (*.xlsx) or inputting in the configure parameter page. In the following configure parameter should be inputted: (1) box parameter (including center of the box(*x*(− 18.1), *y*(17.8), *z*(1.1)) and the size of the box (*x*(23.3), *y*(23.1), *z*(21))) (Additional file 1: Fig. S1B). In addition, their molecular docking pocket is shown in Fig. 7 and (2) the name of the receptor and ligand. After clicking “Perform Calculation”, an ID number is generated for the submitted job. When the calculation is finished, a notification email will be sent to the user's mailbox. The calculation results and output files can be downloaded by clicking "Download" in the Autodock Vina model.

The output file is an MT-CalB-ligands-allFile.zip file which includes input_file, output_file, parameter file and result file: (1) the input_file includes files in pdb format for receptor and ligands; (2) the output_file includes rec.pdbqt, lig.pdb, lig.pdbqt, lig_out.pdbqt, lig_out_*.pdbqt, lig_out_*.mol2, lig_babel.mol2; (3) the parameter file includes configure.json and configure.xlsx; and (4) the result file includes vina_**.log, vina_**.conf, vina.xlsx, vina_score.xlsx.

**Fig. 8:**
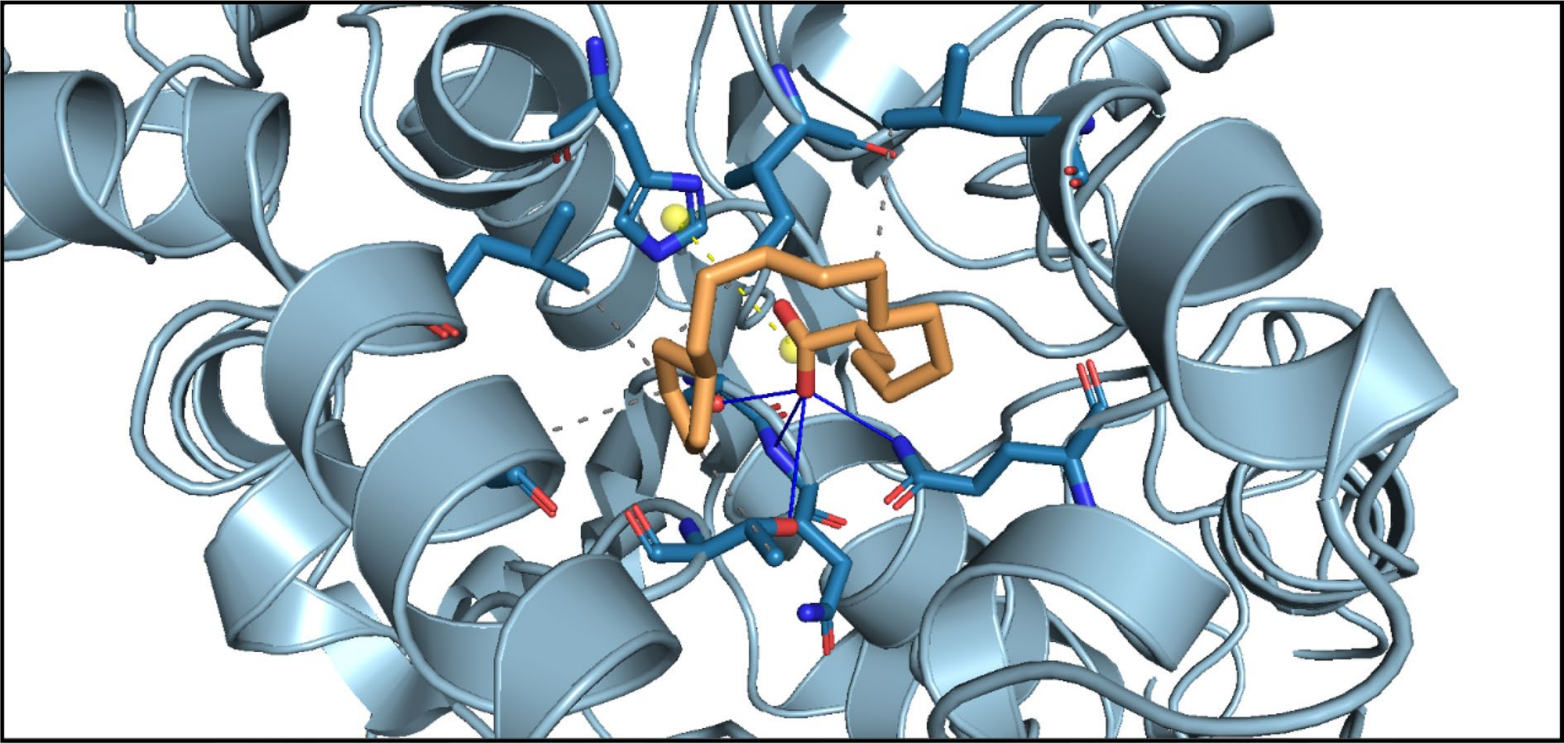
Protein–ligand interactions of MT-CalB with Chaulmoogric acid

The results are showed in Additional file 1: Table S3. The output docking result files record several data, including ligand name, the best binding affinity and so on. According to the best binding affinity and average binding affinity, Chaulmoogric acid possessed the lowest the best and average binding energy (− 5.8 kcal/mol and − 5.6 kcal/mol), followed by Docosahexaenoic acid (− 5.8 kcal/mol and − 5.43 kcal/mol), and Octadecatetraenoic acid (− 5.8 kcal/mol and − 5.39 kcal/mol). The protein–ligand interactions of MT-CalB with CA are represented in Fig. 8 (using Protein Ligand Interaction Profiler (PLIP) server), highlighting the key residues involved in the interaction. PLIP analysis result shows that Thr560 Leu660 Ile709, Leu798, Ala801 of MT-CalB and CA exhibit hydrophobic interaction, while there are hydrogen bonding interactions between Thr560, Ser625, Gln626, Gln677 and CA, salt bridge between His744 and CA. This MT-CalB-CA complex model was used as initial structure for the following example about gromacs model builder module.

### Building the molecular dynamic simulation model of MT-CalB-substrates model using Gromacs model builder module

The MT-CalB-CA complex is used as a model for gromacs calculation. For example, the input files gmx.zip (including: (1) MT-CalB-CA.gro and MT-CalB-CA.top; (2) posre.itp and posre-CA.itp; and (3) EM.mdp) can be submitted by uploading relative files to the system (Additional file 1: Fig. S2A), and the configure.parameters can be submitted by uploading configure.file (parameter.xlsx) or input in the parameter page (Additional file 1: Fig. S2B). After clicking "Perform Calculation", an ID number is generated for the submitted job. When the calculation is finished, a notification email will be sent to the user's mailbox. The calculation results and output files can be downloaded by clicking "Download" in the gromacs module.

The output file is a *.zip file which includes: (1) files after Energy Minimization (em.edr, em.gro, em.log, em.tpr, em.trr, MT-CalB-CA.top); (2) the mdp file for equilibration (NVT.mdp, NPT.mdp) and production MD process (MD.mdp); and (3) a shell script (gmx.sh) to enable user to run equilibration and production MD process on their own computer.

**Fig. 9:**
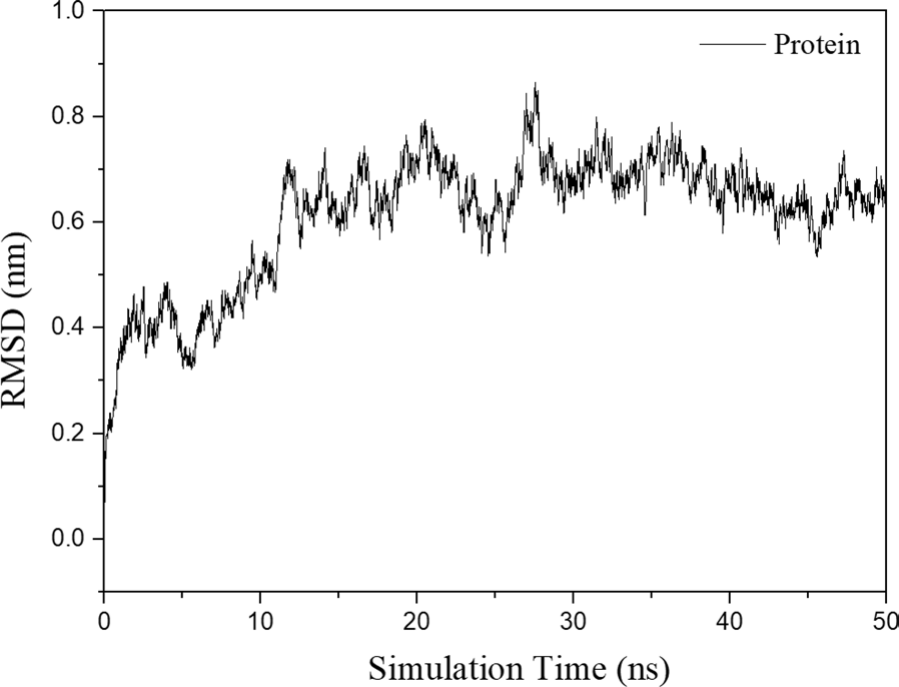
RMSD of the receptor protein

The RMSD of the receptor protein during the molecular dynamic simulation is shown in Fig. 9. In the complex model, RMSD of MT-CalB-CA are about 0.53–0.80 nm, indicating that protein structures were equilibrium during the simulation.

**Fig. 10:**
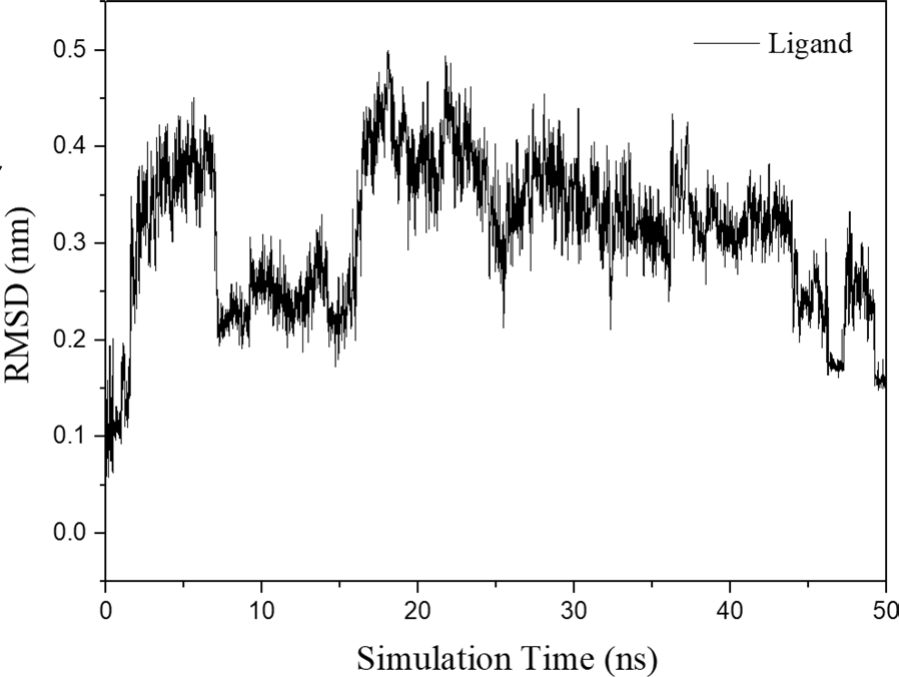
RMSD of the ligand

The RMSD of the CA-ligand during the molecular dynamic simulation is shown in Fig. 10. During the simulation time, RMSD of CA ranged from 0.18 to 0.44 nm, indicating that ligand structure was equilibrium during the simulation.

### G_MMPBSA analysis of MT-CalB-substrates complex using G_MMPBSA module

The Gromacs trajectory of MT-CalB-CA is used as an example for g_mmpbsa module calculation. The input files mmpbsa.zip (including MT-CalB-CA.tpr, MT-CalB-CA.xtc and MT-CalB-CA.ndx) can be submitted by uploading relative files to the system (Additional file 1: Fig. S3A), and the configure parameters can be submitted by uploading configure file (parameter.xlsx) or inputting in the configure parameter page (Additional file 1: Fig. S3B). After clicking "Perform Calculation", an ID number is generated for the submitted job. When the calculation is finished, a notification email will be sent to the user's mailbox. The calculation results and output files can be downloaded by clicking "Download" in the g_mmpbsa model. The output file is an allFile.zip file which includes: input_file, output_file, parameter file and result file. The parameter file includes: configure.xlsx and configure.json. The output_file includes: energy_MM.xvg, polar.xvg, apolar.xvg, contrib_MM.dat, contrib_pol.dat, contrib_apol.dat, energymapin.dat, final_contrib_energy.dat, full_energy.dat and summary_energy.dat. In addition, these above dates are all inserted into an excel file (g_mmpbsa_analysis.xlsx).

**Fig. 11:**
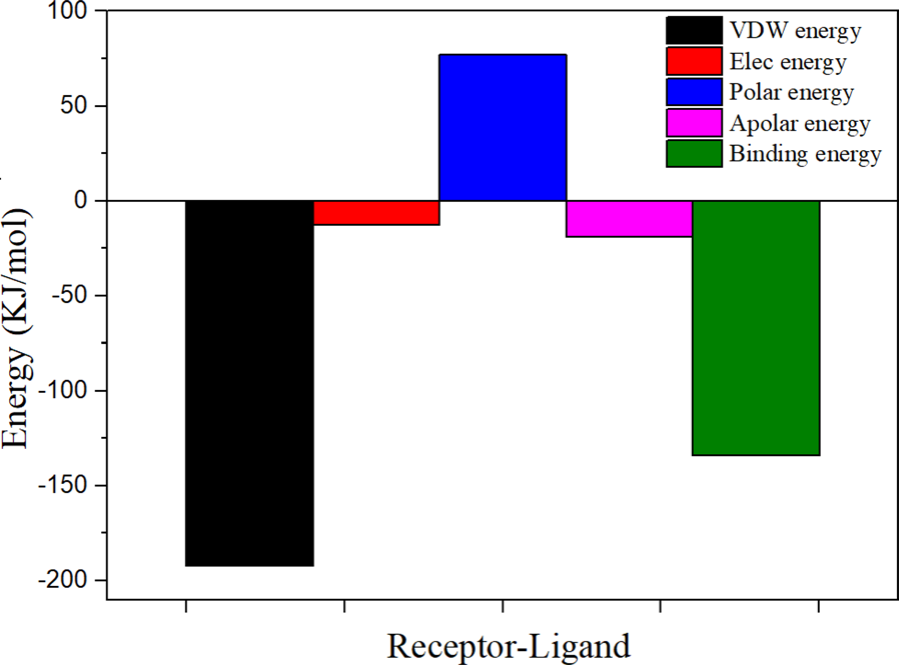
Total energy analysis for the receptor–ligand complex

**Fig. 12:**
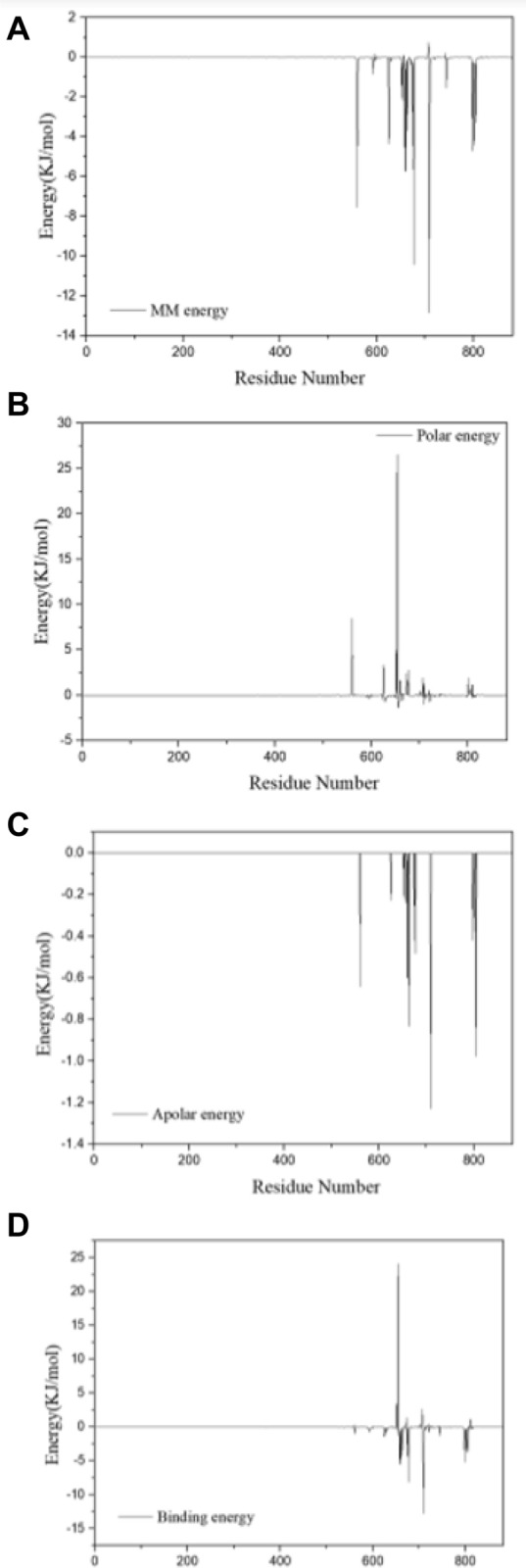
Residual energy contribution analysis of receptor–ligand model

According to the MM-PBSA analysis results (Fig. 11), the Van Der Waals (VDW) interaction and electrostatic interaction are − 179.618 kJ/mol and − 12.618 kJ/mol, respectively. This indicates that VDW interaction plays the key role between MT-CalB with CA. Figure 12 ((A) MM energy contribution (B) Polar energy contribution (C) Apolar energy contribution (D) Binding energy contribution) shows the primary interaction residues between MT-CalB with CA. The polar interactions are observed between CA and Asp654, Thr560, Ala652, Ser625 of MT-CalB. The apolar interactions are observed between CA and Ile709, Ile805, Leu664, Thr560, Leu660 of MT-CalB. The VDW apolar interactions are observed between CA and Ile709, Gln677, Thr560, Leu660, Leu798, Thr658, Val674, Ala801, Ser625, Ala802 of MT-CalB. The results of hydrogen bond interaction analyzed by PLIP are consistent. Ile709, Gln677, Thr658, Leu798 Leu660, Leu664, Val674 are significant contributions to the binding energy residues. These residues are the main binding sites of MT-CalB and CA, and their binding with CA replace hydrogen bonds with water. The results of hydrophobicity are consistent with those of PLIP analysis.

### Experimental verification of design results for MT-CalB

**Fig. 13:**
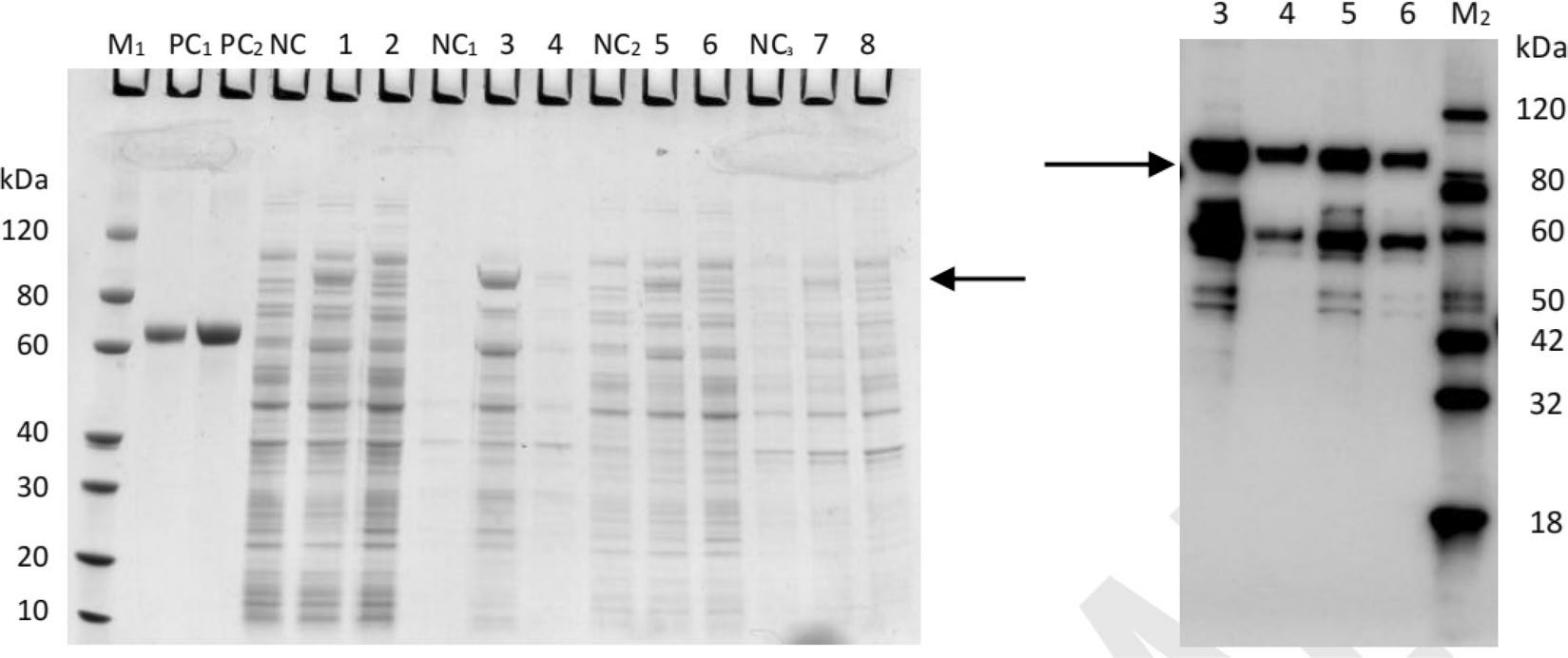
SDS-PAGE result (left) and western blot (right) of MT-CalB. Lane M_1_: protein marker; Lane M_2_: western blot Protein marker; Lane PC_1_: BSA (1 μg); Lane PC_2_: BSA (2 μg); Lane NC: uninduced whole cell; Lane 1: whole cells were induced at 26 oC for 16 h; Lane 2: whole cells were induced at 15 oC for 16 h; Lane NC_1_: uninduced pericytoplasm; Lane 3: periplasmic cells were induced at 26 oC for 16 h; Lane 4: pericytoplasm was induced at 15 oC for 16 h; Lane NC_2_: uninduced supernatant of cell lysis; Lane 5: induced cell lysis supernatant at 26 oC for 16 h; Lane 6: cell lysis supernatant was induced at 15 °C for 16 h; Lane NC_3_: uninduced cell lysis precipitation; Lane 7: 26 °C Induced cell lysis precipitation for 16 h; Lane 8: induced cell lysis precipitation for 16 h at 15 °C

In this study, to increase the soluble expression of CalB in *E. coli*, a number of signal peptides, including mSpA, MBP-TrxA (MT), Carbohydrate Binding Domain (CBD), etc., were fused at N-terminal of CalB. Only MT fused CalB (MT-CalB) could be successfully expressed in *E. coli*. The yield of enzyme activity at optimal fermentation condition were 2085.57 U/L. The SDS-PAGE result is displayed in Fig. 13. The bands belonging to MT-CalB can be observed in the samples of periplasmic cells (lane 3), pericytoplasm (lane 4), induced cell lysis supernatant (lane 5) and cell lysis supernatant (lane 6) with the molecular weight at 93.2 kDa, indicating the successful expression of the MT-CalB.

## Conclusions

In conclusion, an online server with a user-friendly interface has been established by our group, which is constructed for the following purposes: (1) predicting the binding modes between receptor and small molecules using modeller and Auto dock Vina as docking engines; (2) building the gromacs model for dynamic simulation of receptor and ligand complex; and (3) performing the Molecular Mechanics/Poisson–Boltzmann Surface Area (G_MMPBSA) analysis module for receptor–ligand binding affinity analysis. MT-CalB-CA hopes to use it in the fields of biomedicine, energy, and chemical industry. Through this online server, the interaction between MT-CalB and CA, RMSD, the number of hydrogen bonds and Solvent Accessible Surface Area (SASA), and its stability in organic solvents are analyzed. The calculation results show that after MT-CalB interacts with CA, its structural rationality and stability are maintained. Finally, we also verifies through experiments that MT-CalB has better thermal stability and storage stability. The authors hope that the present web server will become a useful tool for enzyme design and computational biology.

### Supplementary Information

**Additional file 1**: **Fig. S1**. Presentation of Vina module (A) Input file user interface; (B) Configure parameter user interface. **Fig. S2**. Presentation of Gromacs module (A) Input file user interface; (B) Configure parameter user interface. **Fig. S3**. Presentation of g_mmpbsa module (A) Input file user interface; (B) configure parameter user interface. **Fig. S4**. The quicklook of each module user what kind of file should be provided. **Table S1**. The whole protein sequence of MT-CalB. **Table S2**. The list of ligands from Pubchem. **Table S3.** The docking result of MT-CalB with ligands.

## Data Availability

All data generated or analyzed during this study are included in this article.

## Abbreviations

CA: Chaulmoogric acid
*CalB*: *Candida antarctica* Lipase B
CBD: Carbohydrate binding domain
*E. coil*: *Escherichia coli*
DS: Discovery studio
GMX: GROningen MAchine for Chemical Simulations
IPTG: Isopropyl β-d-1-thiogalactopyranoside
LB: Luria–Bertani
MBP: Maltose binding protein
MD: Molecule dynamics
MMPBSA: Molecular mechanics Poisson–Boltzmann surface area
*mSpA*: *Mycobacterium smegmatis* Porin A
MT-CalB: MBP-TrxA-CalB
NPT: Constant number of particles (N), pressure (P), and temperature(T)
NAMD: NAnoscale molecular dynamics
NVT: Constant number of particles (N), volume (V), and temperature (T)
PLIP: Protein ligand interaction profiler
*pNA*: *p*-Nitroaniline
RMSD: Root mean square deviation
SASA: Solvent accessible surface area
SDS-PAGE: Sodium dodecyl sulfate polyacrylamide gel electrophoresis
TrxA: Thioredoxin A
VDW: Van der Waals
