# Supplementary material for: Atomevo: a web server combining protein modelling, docking, molecular dynamic simulation and MMPBSA analysis of Candida antarctica lipase B (CalB) fusion protein
Source: Bioresour Bioprocess. 2022 May 13;9(1):53. doi: 10.1186/s40643-022-00546-y (PMC10991163; doi:10.1186/s40643-022-00546-y)
Supplement: Supplementary file 1 — Additional file 1: Fig. S1. Presentation of Vina module (A) Input file user interface; (B) Configure parameter user interface. Fig. S2. Presentation of Gromacs module (A) Input file user interface; (B) Configure parameter user interface. Fig. S3. Presentation of g_mmpbsa module (A) Input file user interface; (B) configure parameter user interface. Fig. S4. The quicklook of each module user what kind of file should be provided. Table S1. The whole protein sequence of MT-CalB. Table S2. The list of ligands from Pubchem. Table S3. The docking result of MT-CalB with ligands. [file 40643_2022_546_MOESM1_ESM.docx]

**Supplementary figures**


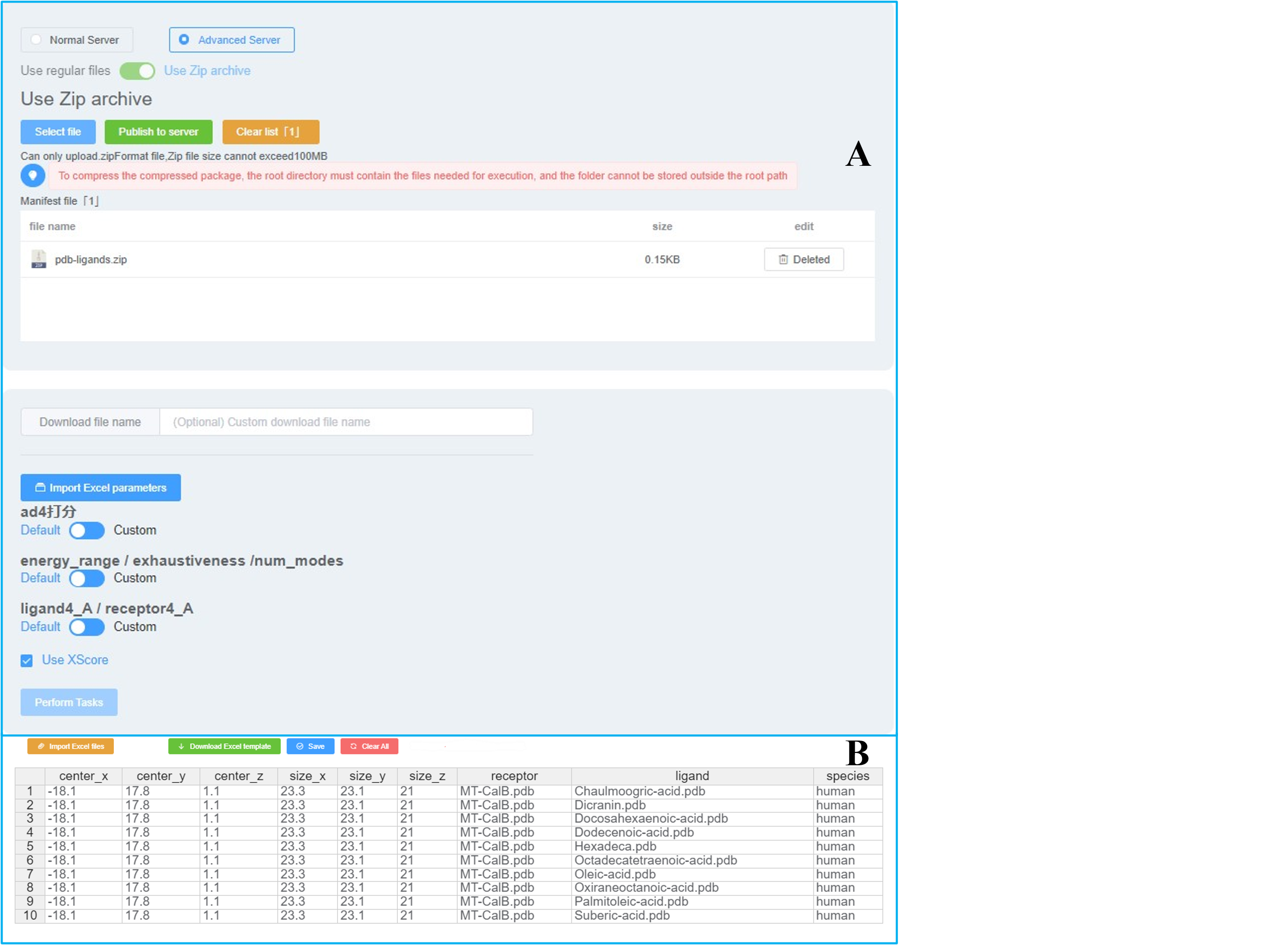


**Figure S1. Presentation of Vina module (A) Input file user interface; (B) Configure parameter user interface**


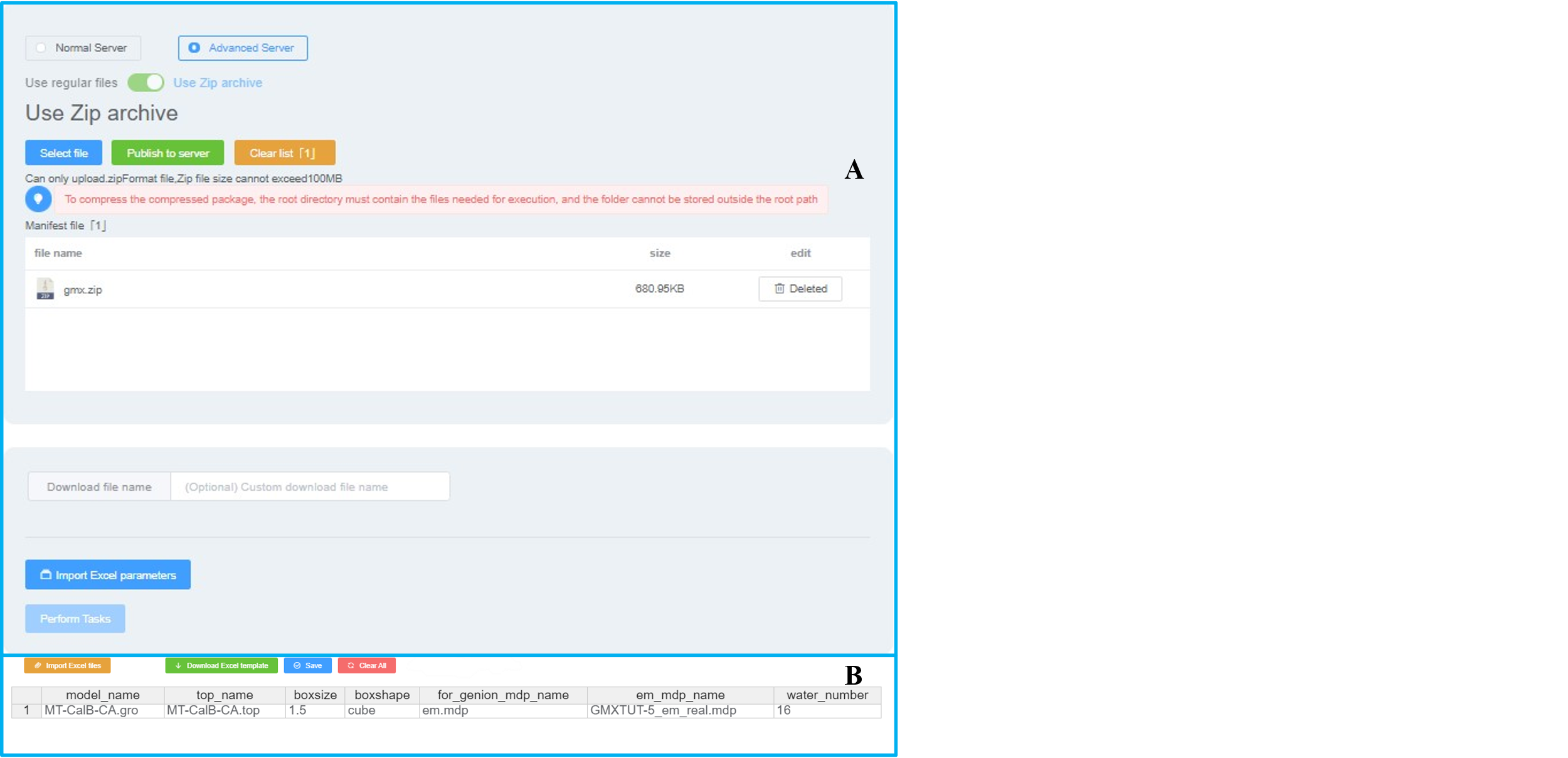


**Figure S2. Presentation of Gromacs module (A) Input file user interface; (B) Configure parameter user interface**


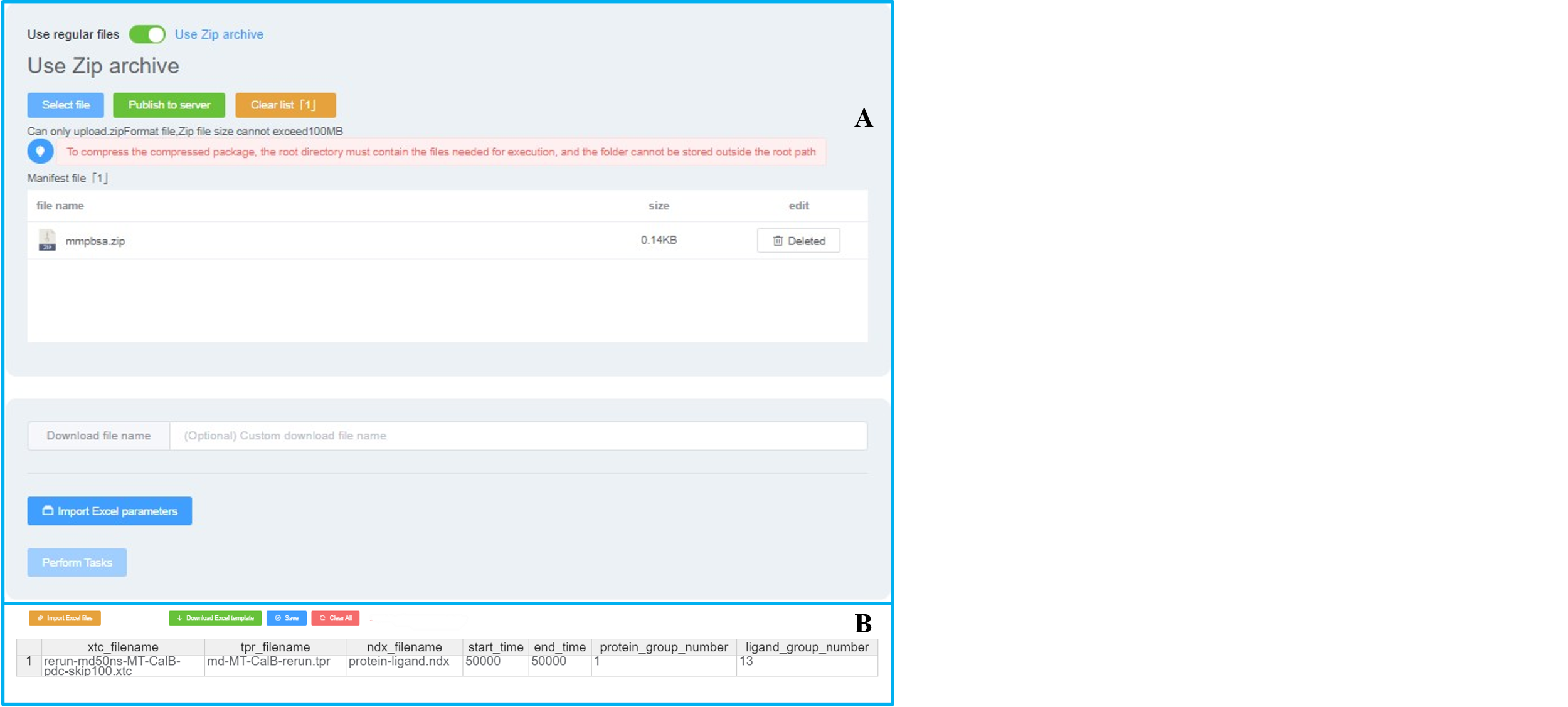


**Figure S3. Presentation of g_mmpbsa module (A) Input file user interface; (B) configure parameter user interface.**


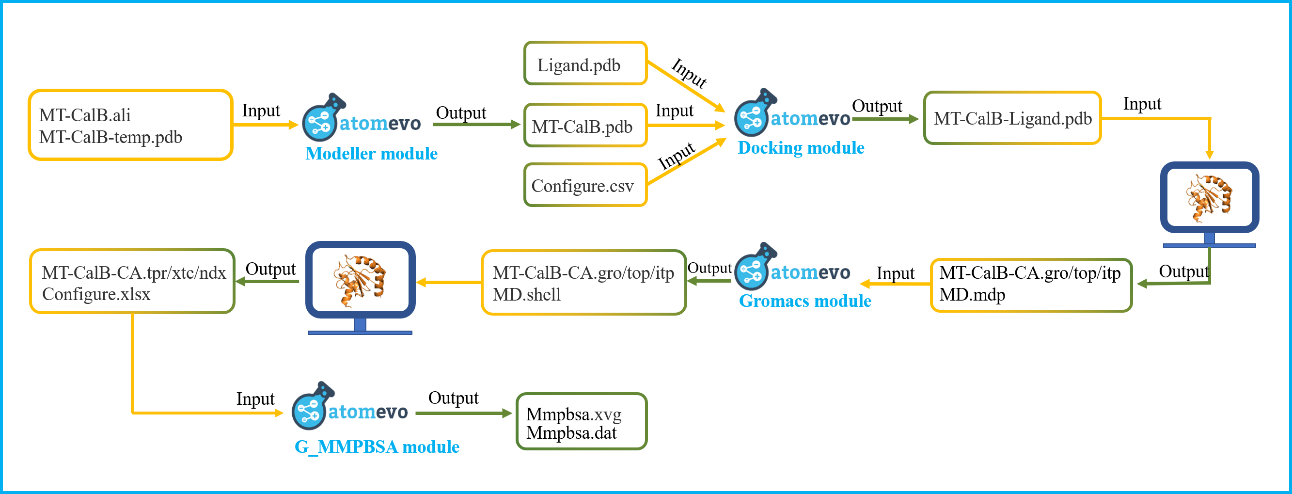


**Figure S4. The quicklook of each module user what kind of file should be provided**

**Supplementary tables**

| **Protein**  **Name** | **MT-CalB** |
| --- | --- |
| **Protein Sequence** | MGSHHHHHHMKIEEGKLVIWINGDKGYNGLAEVGKKFEKDTGIKVTVEHPDKL  EEKFPQVAATGDGPDIIFWAHDRFGGYAQSGLLAEITPDKAFQDKLYPFTWDAVR  YNGKLIAYPIAVEALSLIYNKDLLPNPPKTWEEIPALDKELKAKGKSALMFNLQE  PYFTWPLIAADGGYAFKYENGKYDIKDVGVDNAGAKAGLTFLVDLIKNKHMNA  DTDYSIAEAAFNKGETAMTINGPWAWSNIDTSKVNYGVTVLPTFKGQPSKPFVG  VLSAGINAASPNKELAKEFLENYLLTDEGLEAVNKDKPLGAVALKSYEEELVKD  PRIAATMENAQKGEIMPNIPQMSAFWYAVRTAVINAASGRQTVDEALKDAQTPL  EGGGGSGGGGSMSDKIIHLTDDSFDTDVLKADGAILVDFWAEWCGPCKMIAPIL  DEIADEYQGKLTVAKLNIDQNPGTAPKYGIRGIPTLLLFKNGEVAATKVGALSKG  QLKEFLDANLALVPRGSSMGGGGGSGGGGSQDPLPSGSDPAFSQPKSVLDAGLT CQGASPSSVSKPILLVPGTGTTGPQSFDSNWIPLSTQLGYTPCWISPPPFMLNDTQ  VNTEYMVNAITALYAGSGNNKLPVLTWSQGGLVAQWGLTFFPSIRSKVDRLMAF  APDYKGTVLAGPLDALAVSAPSVWQQTTGSALTTALRNAGGLTQIVPTTNLYSA  TDEIVQPQVSNSPLDSSYLFNGKNVQAQAVCGPLFVIDHAGSLTSQFSYVVGRSA  LRSTTGQARSADYGITDCNPLPANDLTPEQKVAAAALLAPAAAAIVAGPKQNCEP  DLMPYARPFAVGKRTCSGIVTPPNSSSARLQVDKLGSGSGGGGSGGGGSGSAHIV  MVDAYKPTKAAA |

**Table S1. The whole protein sequence of MT-CalB**

| **Ligand**  **Name** | **PubChem**  **ID** | **Molecular**  **Formula** |
| --- | --- | --- |
| Chaulmoogric acid | 72853 | C_18_H_32_O_2_ |
| Dicranin | 44584408 | C_18_H_26_O_2_ |
| Docosahexaenoic acid | 445580 | C_22_H_32_O_2_ |
| Dodecenoic acid | 125207 | C_12_H_22_O_2_ |
| Hexadeca | 2826712 | C_16_H_26_O_2_ |
| Octadecatetraenoic acid | 5312915 | C_18_H_26_O_3_ |
| Oleic acid | 445639 | C_16_H_34_O_2_ |
| Oxiraneoctanoic acid | 1929 | C_18_H_32_O_3_ |
| Palmitoleic acid | 445638 | C_16_H_30_O_2_ |
| Suberic acid | 10457 | C_8_H_14_O_4_ |

**Table S2. The list of ligands from Pubchem**

| **Ligand**  **Name** | **VDM** | **HB** | **HP** | **HM** | **HS** | **RT** | **Score** | **HP**  **SCORE** | **HM**  **SCORE** | **HS**  **SCORE** | **Best Binding**  **Affinity(kcal/mol)** | **Average Binding**  **Affinity(kcal/mol)** |
| --- | --- | --- | --- | --- | --- | --- | --- | --- | --- | --- | --- | --- |
| Chaulmoogric acid | 467.5 | 1.7 | 139.6 | 5.85 | 289.3 | 13 | 5.93 | 5.87 | 6.62 | 5.31 | -5.8 | -5.6 |
| Dicranin | 469.2 | 1.5 | 142.5 | 6.26 | 323.9 | 16 | 5.78 | 5.7 | 6.46 | 5.17 | -5.7 | -5.57 |
| Docosahexaenoic acid | 526.3 | 1.3 | 174.8 | 7.69 | 334.4 | 20 | 5.96 | 5.97 | 6.84 | 5.06 | -5.8 | -5.43 |
| Dodecenoic acid | 348.2 | 1.4 | 91.4 | 3.75 | 219.4 | 10 | 5.17 | 5.12 | 5.58 | 4.8 | -4.9 | -4.78 |
| Hexadeca | 353.6 | 1.2 | 88.9 | 4.11 | 236.3 | 10 | 5.23 | 5.11 | 5.72 | 4.87 | -5.2 | -4.99 |
| Octadecatetraenoic acid | 475 | 2.3 | 122.7 | 5.9 | 261 | 16 | 5.67 | 5.59 | 6.43 | 5.00 | -5.8 | -5.39 |
| Oleic acid | 452.9 | 1.6 | 141.1 | 6.26 | 288.3 | 16 | 5.67 | 5.63 | 6.41 | 4.97 | -5.8 | -5.29 |
| Oxiraneoctanoic acid | 489.5 | 2.6 | 112.8 | 5.54 | 273.9 | 15 | 5.7 | 5.58 | 6.37 | 5.15 | -5.7 | -5.63 |
| Palmitoleic acid | 447.8 | 0.9 | 125.3 | 5.54 | 311 | 14 | 5.65 | 5.55 | 6.23 | 5.17 | -5.7 | -5.43 |
| Suberic acid | 314.6 | 2.7 | 46.4 | 2.15 | 127.2 | 7 | 4.91 | 4.83 | 5.25 | 4.66 | -5.1 | -4.62 |

**Table S3. The docking result of MT-CalB with ligands**
